# Resolving ionic spectra of lead-halide perovskites to the nanometer

**DOI:** 10.1039/d5nr04126k

**Published:** 2026-03-09

**Authors:** Lukas D. Ćavar, Emilia R. Schütz, Yenal Yalçinkaya, Constantin Bach, Carsten Deibel, Lukas Schmidt-Mende, Stefan A. L. Weber

**Affiliations:** a Department of Physics, Johannes Gutenberg University Germany Stefan.Weber@ipv.uni-stuttgart.de; b Max Planck Institut für Polymerforschung Germany; c Institut für Physik, Technische Universität Chemnitz Germany; d Department of Physics, Universität Konstanz Germany; e Institut für Photovoltaik, Universität Stuttgart Germany

## Abstract

Ionic defects play a central role in the function and dysfunction of lead-halide perovskite solar cells yet their distribution and redistribution along the heterogenous thin film is poorly-understood. This is due in part to the limited resolution of conventional optical spectroscopy methods with respect to the nanoscale grain length. Meanwhile, finer-resolution (in particular, scanning probe) methods tend to lack the spectroscopic capability to effectively discern distinct ionic contributions. Here we implement a nanoscale spectroscopic method whereby the capacitance between a conductive atomic force microscopy tip and lead-halide perovskite samples is studied as a function of excitation frequency to yield maps of local distributions of ionic defect spectra in the 100 Hz to 10 kHz regime. We utilize a nonnegative matrix factorization algorithm to disentangle linearly independent contributions to the signal and reveal, in triple-cation lead-halide perovskites, a contrast in the concentration and mobility of ionic species (i) between grains and boundaries, and (ii) between grains. We compare spectra obtained over pristine methylamonnium lead iodide microcrystals *versus* a decomposed pellet to attribute part of the perovskite capacitive response to phase-segregated lead iodide and conclude with a tentative assignment of capacitive resonances. The vicinity of the lead-iodide signature to the primary kHz-range ionic signature of the perovskite solar cell, along with the variation of the primary resonance across the sample surface, may in part explain the difficulties in reaching an experimental consensus of the iodide vacancy activation energy.

## Abstract 2

Low-frequency spectroscopy based on phase-modulated electric force microscopy reveals stark variations in the ionic response across triple-cation perovskite thin films, down to a nanometer resolution.

## Graphical abstract

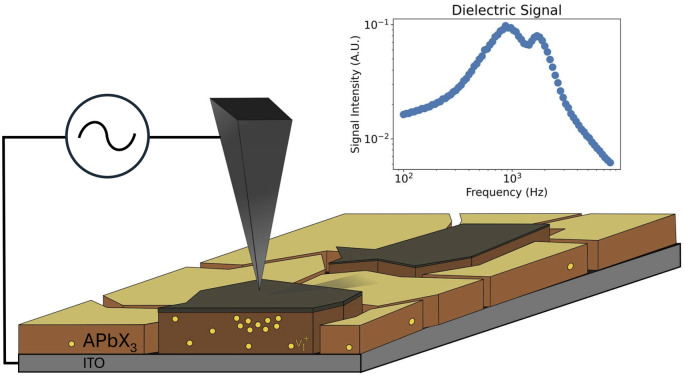

## Introduction

Lead-halide perovskites are a leading candidate among alternative solar technologies for their impressive efficiencies^1–3^ and relatively easy and scalable manufacture *e.g.* by blade-coating.^4,5^ They owe their near-Si performance in part to a highly-tunable lattice and bandgap,^6–8^ defect-tolerance,^9–11^ and low exciton binding energy.^12–14^ But the highly-flexible organometallic lattice also comes with an as-yet untamed ionic degree of freedom: an intrinsically-high population of mobile defects that participates both in the function and dysfunction of perovskite solar cells in a way that is still poorly-understood. This population of defects is thought to be responsible for hystereses,^15–18^ inefficiencies,^19^ and degradation pathways.^20–22^

We are now approaching consensus as to the identity of the relevant ionic players. Element-specific energy-dispersive X-ray measurements,^20,21,23^ electrochemical impedance spectroscopy,^24,25^ nuclear magnetic resonance, tracer diffusion, reaction cell experiments,^26^ as well as first-principles calculations^27–29^ all seem to suggest that the iodide vacancy is the primary mobile species in organic lead-halide perovskites. Migration of the organic cation (in particular methylammonium) is also reported,^23,30,31^ particularly at elevated temperatures, but it is believed to play a less dominant role in cell function. In any case, the values of the key dynamic quantities (activation energies, concentrations) necessary to functionalize the perovskite ionic degree of freedom are far from clear – there is, for example, some controversy in the value of the iodide vacancy activation energy in methylammonium lead iodide, both in experiment^27,32–34^ and in theory.^35^ Response to operating conditions (*e.g.* illumination) further muddies the picture.

As does the nanoscale heterogeneity of the perovskite thin film, as well as the fine dependence of even its bulk properties on the method and parameters of sample preparation. How are ionic defects distributed across the solar cell? And how do they redistribute, either as part of regular photocell operation, or over the course of its degradation? How do we properly engineer the film and tailor its morphology and bulk properties to a scalable photovoltaic application? These questions will not be conclusively answered without nanoscale imaging.

Whereas conventional optical methods are diffraction-limited to several times the relevant length-scales of the perovskite thin film, atomic force microscopy (AFM) is limited only by probe sharpness and by the range of the forces investigated. Consequently, AFM routinely exceeds the resolution needed to resolve grains, grain boundaries, and the associated local variations in the electrical properties of the thin film. This has allowed for local measurements of conductivity,^20,23,36^ capacitance,^37,38^ surface potential difference,^39,40^ and a wide range of other properties over an even wider range of perovskite systems.

By the implementation of a spectroscopic phase-modulation mode (outlined in detail below), we extend the list of AFM capabilities applied to lead-halide perovskites to include the measurement of local ionic response spectra. This method provides rich data as to the relative mobilities and concentrations of ionic defects present in the thin film, and allows for cross-correlation not only with sample morphology, but also with the manifold other AFM modes available on the same device – including Kelvin Probe Force Microscopy (KPFM) and others.

In this work we outline our implementation of the phase-modulated dielectric nanospectroscopy (PM-DNS) mode of measurement, which is based on the method developed by Crider, Miccio, and Schwartz,^41–43^ and similar to the frequency-modulated method by Corrales.^44^ We subsequently apply it to a diverse assortment of lead-halide perovskites as a proof of concept, thereby resolving a finely-varying spatial and spectral contrast that is otherwise washed out in macroscopic measurements. By comparing methylammonium lead iodide (MAPI) to triple-cation perovskite thin films, and pristine to degraded and recrystallized films, we are able to reach a tentative assignment of capacitive responses and their distribution along the sample surface.

## Method

### Phase-modulated dielectric nanospectroscopy

The objective of the method is to couple some measurable aspect of an atomic force microscope (AFM) cantilever's oscillatory motion to the local dielectric properties of the sample beneath it, ideally in such a manner that the electrical drive frequency can be arbitraily modulated. To this end, we note that the electrostatic interaction between a metallic tip and sample is readily modeled as that between two plates of a capacitor separated by a dielectric spacer. The gradient of the capacitive energy immediately yields the electrostatic force that is experienced by the cantilever, which assumes the form:

1 *F*_C_ = ½*C*′(*z*)*V*^2^

with *C*′(*z*) the *z*-gradient of the capacitance at a height *z* and *V* the tip–sample potential difference. The capacitance-gradient term is proportional to the dielectric constant of the spacer. It also has a complicated *z*-dependence arising from the tip–sample geometry, including contributions from the tip apex, cone, chip, and so on. To simplify these contributions, we may Taylor-expand the capacitance-gradient about the cantilever's equilibrium *z*_0_ to obtain:2
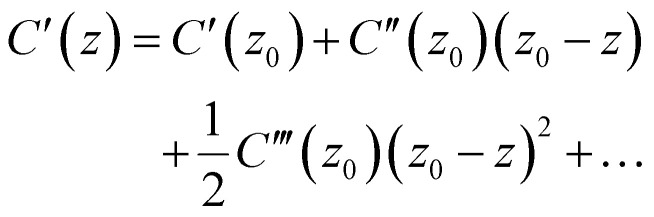


The second term of the Taylor expansion is linear in (*z*_0_ − *z*) and gives rise to a linear electrostatic force. Considered along with the elastic spring force acting on the cantilever, we can see that this particular force gradient effectively stiffens the cantilever:3
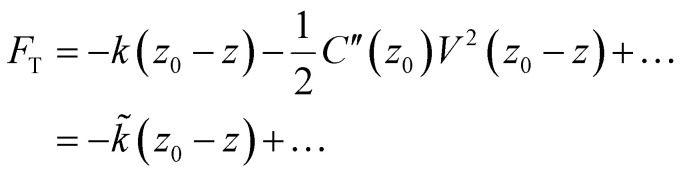
with *F*_T_ the total force acting on the cantilever and *k̃* an effective spring constant.

Insofar as the spring constant determines the resonances of the mechanical cantilever, we can think of ½*C*″(*z*_0_)*V*^2^ as a detuning term. For a cantilever in tapping mode, which is mechanically excited near its first resonance at a fixed frequency *ω*_m_, it can be shown that detuning leads to an additional phase shift between the mechanical drive and the actual mechanical oscillation. In the small-excitation limit, this phase shift is linearly proportional to *δk* = −½*C*″(*z*_0_)*V*^2^ the effective cantilever stiffening and hence depends on the second capacitance-gradient and the square of the tip–sample voltage.

Though many factors affect the phase of mechanical oscillation, the electrostatic stiffening is readily modulated by applying an AC voltage between the tip and sample at some frequency *ω*_e_. Provided that this frequency is sufficiently small relative to the mechanical frequency of oscillation, we immediately obtain a corresponding modulation to the mechanical phase of oscillation that can be measured by standard lock-in detection methods. We note that, since the potential difference enters into our force gradient as a square, the phase shift modulated at the first and the second harmonic of the frequency of electrical stimulus. The demodulated amplitude of the second harmonic phase-oscillation is proportional to a great number of factors – including, significantly, tip–sample geometry and area of contact – but its leading frequency-dependence lies exactly in the local sample dielectric constant.^41–43^ Hence the significant challenge of signal quantification is effectively avoided when considering a spectroscopic modality where the signal amplitude is studied at a fixed position as a function of stimulus frequency *ω*_e_.

The accessible frequency range is set, on the upper end, by the bandwidth of the cantilever's mechanical excitation, and, on the lower end, integration time and prevalence of low-frequency noise. In our implementation, we were routinely able to probe frequencies between 100 Hz and 10 kHz, which was sufficient to capture the primary lead-halide perovskite ionic resonances.

The signal's dependence not on the capacitance itself but on its second derivative not only obviates the need to correct for the large stray signals routinely encountered in conventional capacitive AFM measurement,^45^ but also yields an intrinsically more local signal owing to the singularity-strengthening effect of the derivative. The effectively-enhanced resolution allows access to even the nanoscale regime of grain boundaries in lead-halide perovskites, which is particularly relevant in this study.

### Measurement procedure

Thin films were prepared as described in the SI and subsequently stored and measured in a nitrogen atmosphere glovebox. Once fixed to the sample holder, a nick was scratched into the perovskite, exposing the conductive substrate beneath. A grounded metallic probe was affixed to the surface of the thin film in the direct vicinity of the nick, and the area was covered in silver paste to ensure an electrical connection.

The measurement scheme as implemented here requires two demodulation steps: (i) first, the demodulation of the mechanical deflection relative to its reference drive to obtain the mechanical phase of oscillation, then (ii) the demodulation of the mechanical phase *versus* the electrical stimulus frequency. The first demodulation step was achieved by a Zurich Instruments HF2LI lock-in amplifier which was synchronized to the mechanical drive of an MFP 3D AFM (Asylum Research, Oxford Instruments) to serve as phase reference. The demodulated phase was further fed to a Zurich Instruments MFLI lock-in amplifier, which was also used to generate the electrical excitation between tip and sample: the system was grounded through the sample substrate and an AC voltage was routed through the AFM control box directly to the metallic tip. Finally, the mechanical phase was demodulated by the MFLI with respect to the second harmonic of the electrical excitation to obtain the amplitude of the capacitance signal at a particular frequency.

A measurement is carried out in surface dwell height feedback mode: (i) the tip approaches the sample to a set mechanical amplitude, (iia) height feedback on the mechanical amplitude maintains a fixed tip–sample distance, (iib) concurrently, the frequency of electrical stimulus is swept bidirectionally and the resulting periodic phase shift is demodulated to obtain a capacitive spectrum for that pixel, (iii) the tip retracts and moves to the next pixel. The process is repeated at many pixels to obtain a hyperspectral image.

## Results

### Triple-cation perovskites

The partial substitution of formamidinium, cesium, and bromine into the methylammonium lead iodide (MAPI) lattice allows for chemical tuning of the resulting bandgap along with enhanced lattice stability.^46–48^ These so-called ‘triple-cation’ thin films are the most promising of the perovskites from a photovoltaic perspective.^49^

**Fig. 1:**
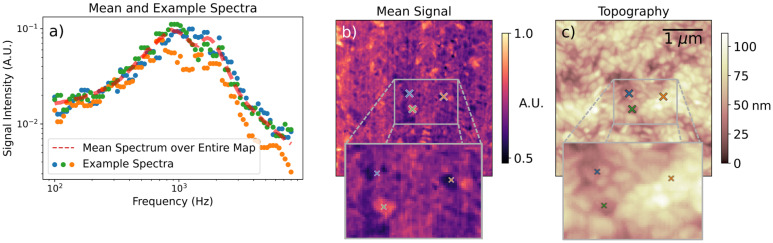
PM-DNS map over a granular triple-cation perovskite thin film. (a) Mean spectrum collected over the entire field of view, along with three particular representative spectra. (b) Mean signal intensity over the extent of the map. Representative pixels marked by color. (c) Topography. Note a granular morphology typical of lead-halide perovskite thin films, an enhancement of the mean signal at grain boundaries, and an enhancement of the mean signal in the bulk of some grains.

The topography of such a thin film is shown in Fig. 1, where we observe a granular film of typical grain size near 100 nm. In the same area we perform a frequency sweep between 100 and 8000 Hz at each individual pixel and record the capacitance signal. Fig. 1b depicts the associated distribution of mean signal intensities: we observe (i) a signal enhancement at grain boundaries, and (ii) a signal enhancement at certain ‘hotspot’ grains. We can use KPFM (see section on Kelvin probe force microscopy) to further study the nature of the hotspot grains: KPFM over the same sample region reveals a correlation between capacitance-enhancement and an enhanced (more negative) surface-potential difference (see the overlay in Fig. S1 for a direct comparison). For the CPD convention used in this manuscript (CPD = *V*_sample_ − *V*_tip_), this is consistent with a higher density of negatively-charged defects in the hotspot grains or a difference in the local work function (which in turn could be due to many factors). Going forward, we will refer to grains that exhibit enhanced ionic response alongside an enhanced CPD as ‘hotspot grains’.

**Fig. 2:**
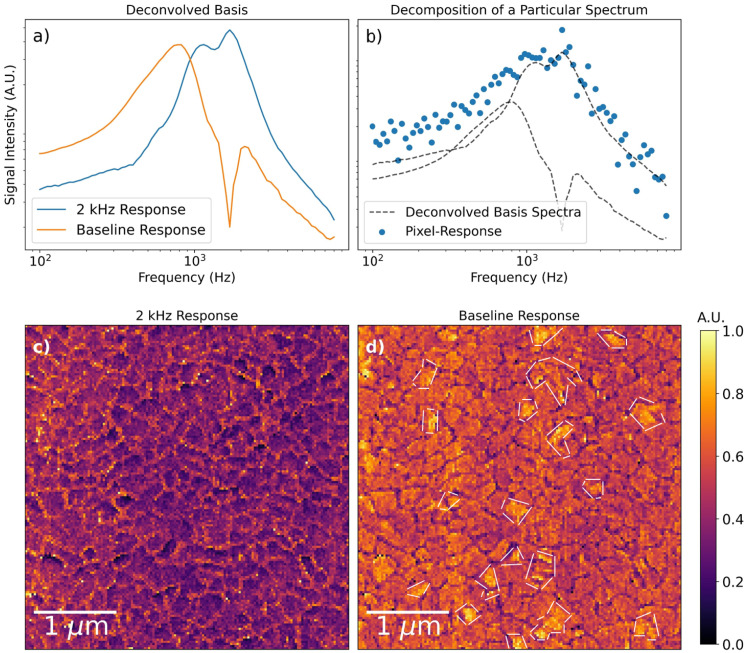
(a) Basis spectra over a triple-cation thin film deconvolved by NMF. (b) Example of a particular spectrum decomposed into a basis of deconvolved spectral components. (c and d) Respective weights of deconvolved basis components across the extent of the map. Hotspot grains identified in Fig. S1 are outlined in white.

Considering now the actual spectra collected at each pixel, we first examine three representative points: a typical grain, a grain boundary, and a hotspot grain. These are reproduced in Fig. 1a along with the mean spectrum over the entire map. It is clear from the mean spectrum that the signal consists of two peaks: one near 1 kHz and another near 2 kHz. It is also clear from the representative spectra that these signals vary across the sample. To study this variation systematically, we employ a nonnegative matrix factorization (NMF) algorithm to project the high-dimensional signal-space into a low-dimensional linear combination of ‘basis spectra’ chosen to minimize the reconstruction error.^50^ The factored basis spectra can then be interpreted as independent underlying physical processes and the weight of that basis spectrum at a particular pixel as the relative prevalence of that process in that place. NMF is routinely used to deconvolve hyperspectral data that contains a linear combination of spectral contributions;^51–53^ it is entirely independent of the underlying physics and makes no assumptions regarding the shape of the data or its basis, which is determined uniquely up to a permutation and scaling factor. In Fig. 2 we apply an NMF decomposition to the dataset depicted in Fig. 1. The deconvolved two-dimensional basis consists of a low-frequency peak and two correlated high-frequency peaks. The spatial distribution of their respective weights reveals that the high-frequency components are most prevalent at grain boundaries, while the low-frequency component is most prevalent in the grains themselves – with additional enhancement at the hotspot grains.

**Fig. 3:**
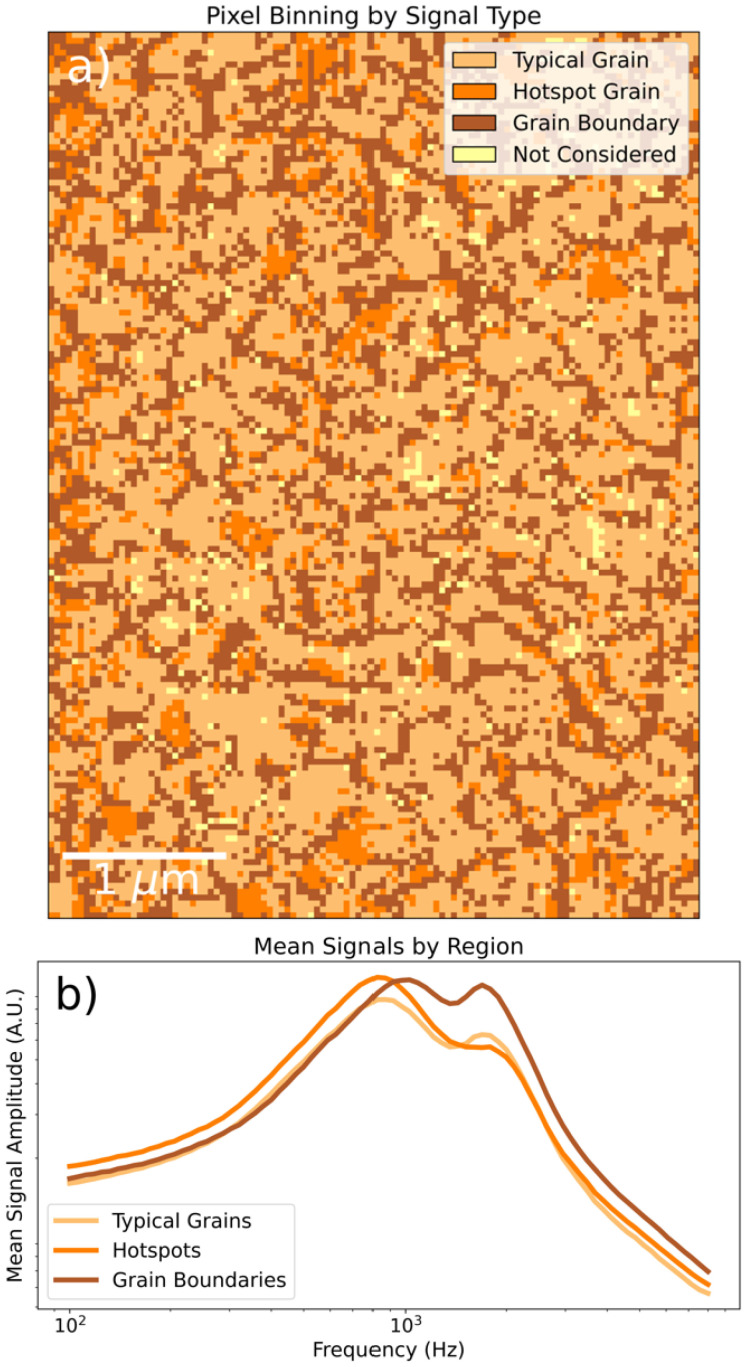
(a) Labeling of points by dominant NMF component. Note the first forty scan lines are excluded due to ultraslow hysteresis. Outlier spectra (with weights lying on extreme combinations of percentiles) were not included in the classification, and are labeled as ‘Not Considered’. (b) Mean signals by signal type.

To further analyze the data, we classify individual spectra by their relative weights: whether they are dominated by the high- or low-frequency basis spectrum, or whether their low-frequency component is exceptionally large. This is done by selecting combinations of cutoff values of the NMF weights based on their percentiles to select representative populations of spectra. The classification is reproduced in Fig. 3 along with the mean spectrum by class. The mean spectra clearly reveal that (i) at grain boundaries, the kHz resonance is bluehsifted and the 2 kHz resonance is enhanced, and (ii) at hotspot grains, the kHz resonance is enhanced. This line of analysis is repeated with a smaller field of view over a different region in Fig. S2 and yields consistent results. The smaller scan reveals that the enhanced ionic response is localized to a specific sub-region of the grain that is topographically distinct, often appearing as a raised feature. Additionally, in the closeup image, it appears that the effective grain boundary zone is thicker than in the previous measurement – the bright dielectric halo of the grain boundary seems to persist deeper into the grain. As comparison of topographical measurements (S1 and S3) reveal no significant differences in the grain boundary profile, the effect does not appear to be due to tip blunting but may rather be a result of structural degradation in and about the grain boundary over the course of repeated measurement. The scale and morphology of the CPD contrast also looks significantly different from the fresh film: see Fig. S3, where the CPD within a grain is less homogenous than in Fig. S1, and the CPD contrast is about five times greater in places. We may speculate that, over the course of our first measurement, the sample's ionic landscape was significantly remodeled due to large-scale ion migration and ongoing film degradation. Since a single PM-DNS scan can take upwards of 24 hours, the observed changes in sample morphology reflect the sample degradation over several days of continuous electrical stimulation. Large voltages, such as the ones employed during PM-DNS measurements, have been shown to accelerate degradation,^20,21,54,55^ and it is also conceivable that hotspot grains, if they are a reservoir of additional ionic defects, may be preferential sites of degradation–nucleation, especially near a grain boundary.

The origin of the 1 kHz and 2 kHz resonance peaks observed in triple-cation perovskite thin films is not clear *a priori*. Firstly, macroscopic EIS measurements tend to report kHz-range ionic response only in the presence of additional charge-transport layers.^18,56^ In their absence, and in particular in the half-cell configuration corresponding most closely to our triple-cation perovskite samples, only a very low frequency response is reported.^34^ This discrepancy may be explained by the fact that, although our sample is a half-cell, our experimental geometry is closer to a solar cell than to a coplanar device (as in Schmidt^34^), since the electrode is connected laterally through the perovskite layer to the ITO. If, as some authors suggest,^18,56^ the kHz ionic response is a signature of Debye layer formation, it is plausible that such a geometry would yield the observed signal. The high voltages we employ during measurement (∼2 V *versus* the ∼10 mV routinely used in EIS) may also play a role in provoking the kHz response. Simulations may shed further light on this question.

Secondly, macroscopic studies tend to report either a single peak or a distribution of single peaks varying across the film,^25^ but not two distinct resonances. A possible explanation is that, since macroscopic measurements sample a wide distribution of varying spectra at once, the two features are washed out to resemble a single peak in the resulting measurement.

Since we cannot rely on macroscopic methods to decipher the two peaks in triple-cation perovskite, another strategy would be to repeat our measurement under varying material conditions. By varying the perovskite species, degradation-state, morphology, and sample size, we may obtain enough information for a tentative peak assignment. To this end we also measured spectra over pure MAPbI_3_ micro- and millicrystals in different states of degradation.

## MAPbI_3_

### Pristine microcrystals

**Fig. 4:**
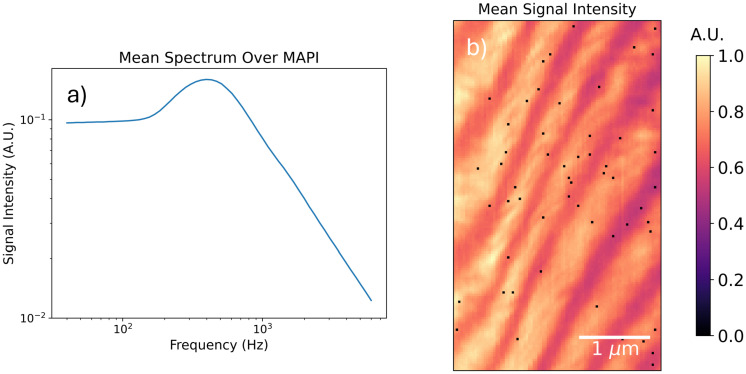
PM-DNS measurement over MAPI twin domains. (a) Average spectrum collected over selected field of view. (b) Mean signal intensity map.

In Fig. 4 we reproduce the average PM-DNS spectrum collected over a pristine MAPI microcrystal grown under confined volume crystallization (see Methods for details). The spatial variation of the signal's mean intensity is also included. We observe a stripelike spatial variation in the signal intensity that does not correspond to any other variation in the spectra; that is to say, the spectra between stripes vary only in their respective intensities, not in their apparent resonance frequency.

**Fig. 5:**
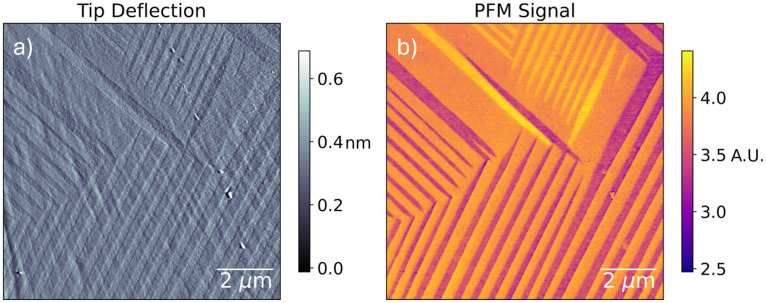
PFM of twin domains in MAPI crystallites grown under confinement. (a) AFM deflection. (b) PFM amplitude signal. The three levels of contrast correspond to three different piezoelectric responses which are attributed to three crystallographic orientations due to the anisotropic piezoelectric response.

Unlike triple-cation perovskites, pure MAPbI_3_ is known to exhibit twinning into alternating domains of crystal orientation in response to strain.^57^ To confirm that the stripe contrast we observe in PM-DNS corresponds to twin domains in our MAPI sample, we perform piezoresponse force microscopy (PFM)^58^ on the sample surface. Over the course of PFM, the tip is brought into contact with the sample and electrically stimulated at a frequency close to the tip's contact resonance. At this particular frequency, the piezoelectric response of the sample and the mechanical system it forms with the tip interferes constructively with itself, leading to a large-amplitude oscillation of the cantilever while the tip is in contact. This oscillation can be demodulated and measured over the extent of the sample surface: variations in the amplitude and phase of the induced oscillation are due to variations in the contact resonance frequency, which, in turn, will correspond to changes in the lattice orientation under the tip due to the anisotropic piezoelectric response of MAPI. Hence the PFM amplitude and phase reveals the twin domain structure of our sample, which is reproduced in Fig. 5. Here we observe a clear three-level PFM contrast, corresponding to three distinct crystallographic orientations. This, along with the relative orientation of domains (70° angles), and their faint topographical contrast suggests out-of-plane twinning,^57^ which is consistent with the high stresses of confined crystal growth.

**Fig. 6:**
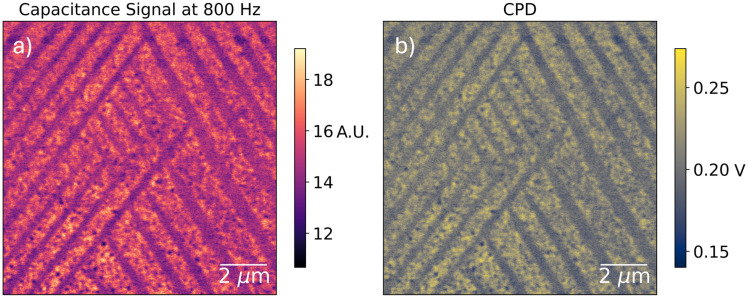
(a) Single-frequency EFM signal measured by PM-EFM over MAPI microcrystals at 800 Hz. This frequency is chosen because it is a balance of high signal intensity (see Fig. 4) and high frequency, and correspondingly low settling time between pixels. (b) Surface potential difference measured by KPFM over the same area. The contrast is nearly identical.

Although PFM reveals the twin domain structure, and it is clear that the stripelike PM-DNS contrast corresponds to different sets of twin domains, it is not immediately clear why twinning should impact the local ionic response. To shed light on the connection, we perform further electrical characterization measurements: namely, KPFM along with correlated single-frequency phase-modulated electric force microscopy (PM-EFM). The latter is just PM-DNS measured at a single frequency of interest and scanned across the surface – allowing for higher-resolution measurement of the local ionic response. Meanwhile, KPFM is a method used to measure variations in the built-in contact potential difference between tip and sample:^59^ a feedback on the mechanical oscillation induced by the capacitive force between tip and sample continuously adjusts an applied bias voltage to cancel the contact potential difference, thereby deducing it across the surface of the sample. As a nanoscale voltmeter, KPFM is sensitive to variations in work function, surface chemistry, and local charge accumulation. By correlating KPFM and PM-EFM over the same scan area we can compare signal contributions from fast and slow components of the electrical system. Such a correlated measurement is reproduced in Fig. 6, where we observe that the contrasts are identical.

The interdomain contrast is explained by one of two things: (i) redistribution of charged defects between twin domains, also reported by previous authors,^60^ or (ii) a work function contrast between crystal faces. Either would explain the CPD contrast. As for the EFM contrast: the ionic response of organometallic semiconductors, and particularly perovskites, is known to vary finely with the prebiasing conditions of the perovskite cell.^61^ The spatial variation in the capacitive signal that we observe here may well be a result of an inhomogenous built-in ‘local prebias’ (originating, for example, in a work function contrast) and cannot be definitively attributed to mere ionic redistribution without a more quantitative study of the MAPI voltage-response.

Neither of these explain the ‘marbling’ pattern observed in bright EFM and KPFM regions: as the individual defects observed are consistent between sequentially-measured KPFM and PM-EFM maps, we cannot attribute them to noise or topographic variations. We must conclude that they are isolated defects, likely on the surface of the MAPI. Since they are confined to only one set twin domains, we may speculate that they are regions where localized surface degradation has begun, and that such degradation nucleates preferentially in the defect-enriched set of twins. Otherwise, since the crystals are grown between polymer-coated plates, polymer residue or surface templating are other likely explanations for these isolated defects.

The mean spectrum collected over the MAPI microcrystals presents a clear peak near 600 Hz. The absolute value of the signal, which is depicted in Fig. 4 and elsewhere, is proportional to the sum of the real and imaginary components of the dielectric response of MAPI. Macroscopic measurements of similar crystals – say, by electrochemical impedance spectroscopy^25^ – are in agreement with our measured microscopic resonance frequency.

### Degraded pellet

In order to further study the effects of sample size and degradation on the PM-DNS hyperspectra, we measure the ionic response over a several-month-old mm-scale MAPI pellet. For the months prior to measurement, the pellet was sealed in a vial under nitrogen atmosphere, and was only exposed to air while mounting the sample.

**Fig. 7:**
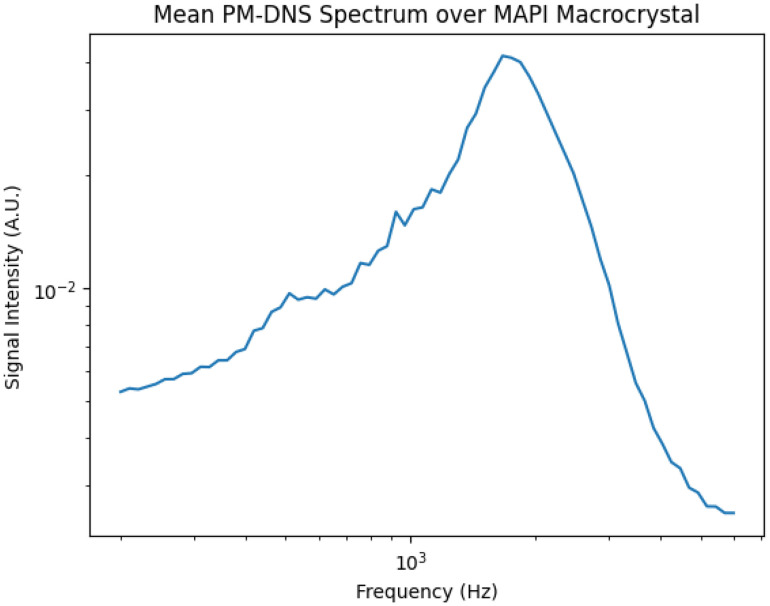
Mean PM-DNS signal obtained over degraded macroscopic MAPI pellet depicted in Fig. S4.

PFM reveals a possible faint twinning signature but overall a rough and visibly-degraded surface: see Fig. S4. Meanwhile, the collected PM-DNS hyperspectrum presents little spatial contrast. The map mean spectrum reveals a faint 600 Hz resonance peak along with a strong 2 kHz peak: see Fig. 7.

## Interpretation

We attribute the 1 kHz and 600 Hz signal peaks to the same defect, the iodide vacancy, presenting different defect mobilities in different lattices. The frequencies observed are in agreement with macroscopic studies of MAPI and triple-cation perovskites^18,25,56^ and the peak blueshift at grain boundaries in the triple-cation sample is consistent with previous time-resolved conductive AFM studies.^20,21^ Similarly, the coincident enhancement of the kHz resonance is in line with various simulations indicating preferential defect accumulation at boundary sites.^28,62^ In the case of MAPI, the fact that the 600 Hz resonance is independent of sample size is further evidence that it is a signature of bulk transport rather than an interfacial phenomenon.

We note that the 2 kHz resonance is greatly enhanced in the degraded MAPI sample and well-localized to the edges of apparent sites of lead-iodide phase-segregation (see Fig. 11). Furthermore, the 2 kHz line is observed in both MAPI and the triple-cation samples, indicating that it is lattice-independent. While lead-iodide itself does not appear to present a clear ionic resonance,^63–65^ we may still attribute the signal to the new interface between the lead-iodide and surrounding perovskite. In any case, we tentatively conclude the 2 kHz response is a signature of degradation, and in particular of local lead-iodide phase-segregation. Since grain boundaries are a region of relative disorder and hence a favoured site for degradation-nucleation,^20,55^ the enhancement of the 2 kHz signature there is not surprising. What is more surprising is the lack of 2 kHz-enhancement at the hotspot grains: previous KPFM studies of similar thin films have suggested the grain-grain CPD contrast is a result of variations in the local lead-iodide content.^66^ This is not consistent with the PM-DNS contrast, where an enhancement of the 1 kHz peak (and *not* the 2 kHz peak) suggests an enhancement of the mobile defect concentration rather than just a local prebiasing effect as discussed for MAPI. Additionally, the fact that the 1 kHz resonance is not visibly blueshifted at hotspots indicates that all grains are structurally similar from the perspective of iodide vacancy transport, and that the origin of defect segregation between grains lies in a facet or other surface contrast rather than a stoichiometric one – on the grounds that a significant stoichiometric contrast would also shift the iodide vacancy resonance. This suggests that the origin of the CPD contrast is either due to degradation on the grain surface, an enhancement of the density of charged defects, or a combination of the two. If this interface contrast can be attributed to a lead-iodide cap, as has been previously suggested, then the nature of the PbI_2_ – perovskite interface must be different on the hotspot grains than at the grain boundaries – in the sense that a degraded grain boundary would play a different role in ion transport than a degraded grain surface.

## Methylamine treatment

A tentative interpretation of the signal allows for an investigation of a more complicated case: triple-cation perovskite thin films recrystallized under the influence of a methylamine (MA) atmosphere. The MA treatment is a promising technique for the potential recycling or upcycling of lead-halide perovskite thin films and has been shown to significantly change the film morphology, nature of grain boundaries, and the distribution of electrical properties.^67,68^ Here we use it to investigate the role of grain morphology and size on local ionic transport, as well as to visualize the sub-granular morphology otherwise inaccessible in nanoscale grains.

**Fig. 8:**
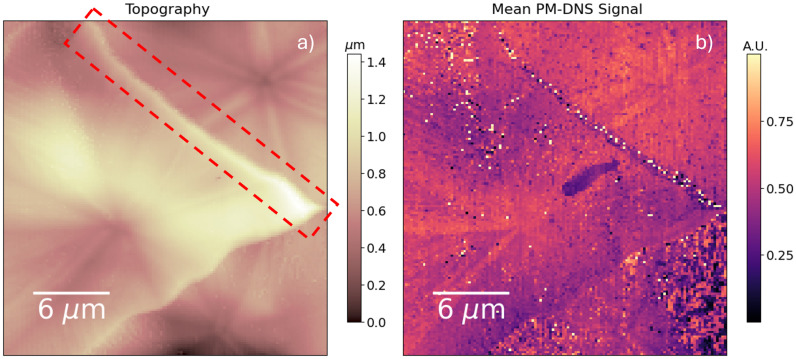
(a) Topography and (b) mean signal intensity measured over a triple-cation perovskite film treated with MA at 73 °C. The “inactive grain boundary” discussed in the main text is outlined in red on the topography. Grains treated at a particularly low temperature tend to grow very large due to the low density of nucleation sites, and occasionally present the visible starlike morphology.

The topography of an MA-treated thin film (see Fig. S5 and Fig. 8) reveals an enormous grain size on the order of microns – nearly two orders of magnitude larger than the untreated film – along with significant topographical contrast. The ionic response, on the other hand, is remarkably homogenous compared to measurements over untreated thin films: see Fig. S5 in the SI. Hence, even though MA-treated grains often present nontrivial subgranular morphology,^67^ the relevant length scale of the ionic landscape remains the grain-scale.

We can influence the grain-scale by varying the temperature of treatment: at lower temperatures, grain nucleation is sparse and slow, leading to larger grain sizes. Particularly low temperatures, however, result in crystallographic inhomogeneities within the grain.^67^Fig. 8 shows such a grain (treated at 73 °C) and the corresponding ionic contrast. A starlike projection from the center of the grains is visible not only in the topography but also in the intensity of the PM-DNS signal. According to previous electron backscatter diffraction measurements of similar treated grains, the alternating star projections correspond to alternating lattice orientations.^67^ Besides the intragrain contrast, we also observe a clear signal decrease along one of the two grain boundaries. The affected region shows an inordinately thick grain boundary (nearly a micron), compared to the ∼10 nm grain boundaries observed in the reference sample. Interestingly, only one of the two imaged grain boundaries shows this anomalous signal – the southern grain boundary is only slightly dimmed at the edges. The dielectric character of a particular grain boundary is likely dependent on the specific recrystallization history of the surrounding grains, as unstoichiometric material is swept out along with the crystallization front to accumulate at specific edges and not at others.

**Fig. 9:**
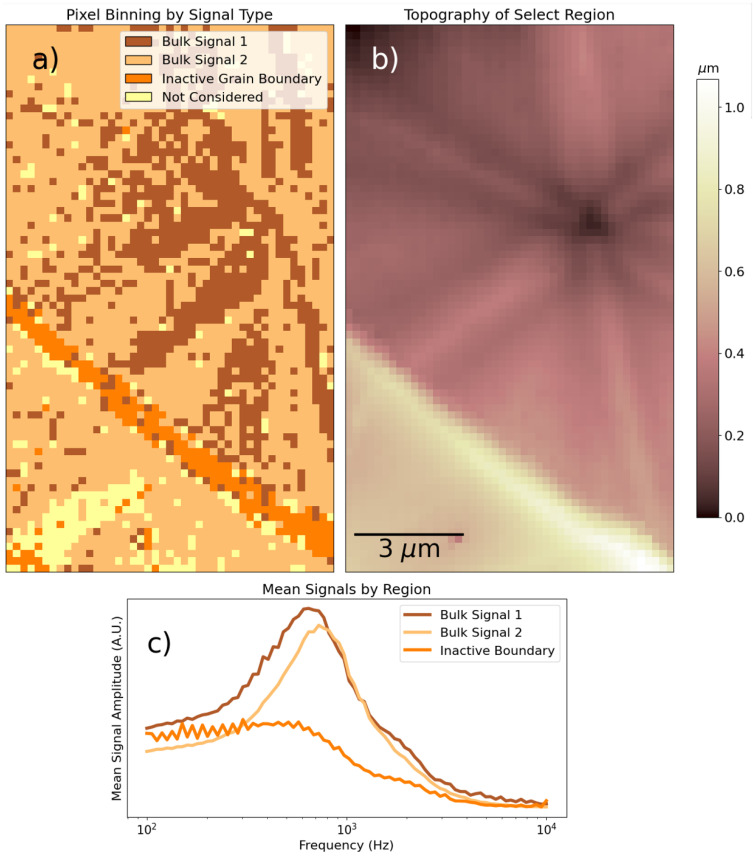
Classification of spectra in Fig. S6 according to dominant NMF component. (a) Labeling of points by classification. (b) Topography of region of interest, zoomed in from Fig. 8. (c) Average spectrum by grouping in (a).

An NMF decomposition reveals the contrast more clearly. It is reproduced in the SI, in Fig. S6. In Fig. 9, we select a part of the measured region, once again group spectra by dominant NMF component, and subsequently average the signal by group. In one set of lattice orientations, we observe a signal reduction and blueshift. The contrast in resonance frequency may be due to an anisotropic ion mobility within the perovskite lattice, or due to structural variations in the alternating pairs of sub-domains. A local pre-biasing effect may also be at play, locally changing the dielectric response along with the perovskite facet contrast. Meanwhile, at the grain boundary, we observe a strong reduction in the dielectric response. Due to its inordinate thickness and the nature of the recrystallization process, we suppose the inactive grain boundary has a lower perovskite content than the grain boundaries in the reference samples.

### Degraded MA-treated triple cation

**Fig. 10:**
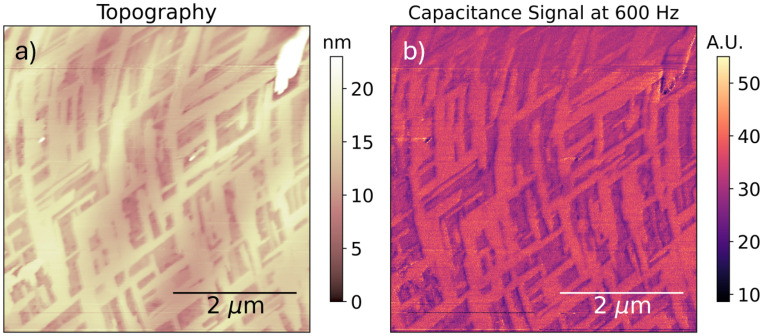
Single-frequency (600 Hz) PM-EFM scan over a particularly-degraded or contaminated MA-treated triple-cation perovskite thin film. (a) Topography. (b) Capacitance signal intensity. Note capacitance is enhanced over the raised features – this is not a geometric effect, but likely a variation in the local dielectric response. It is remarkable that a mere 20 nanometer topographical contrast should coincide with a near-doubling of the capacitance signal.

Finally, we turn to measurements performed over a contaminated or particularly-degraded film of MA-treated triple-cation perovskite. The specific nature of its contamination is not well-understood and is only used to illustrate the possible effect that contamination or advanced degradation can play in the perovskite ionic landscape. This thin film is characterized by a mesh-like texture covering the surface of the sample, which we show in Fig. 10. There we also depict the single-frequency PM-EFM contrast coinciding with the texture, which shows a considerable increase in the local capacitance over the raised mesh features.

**Fig. 11:**
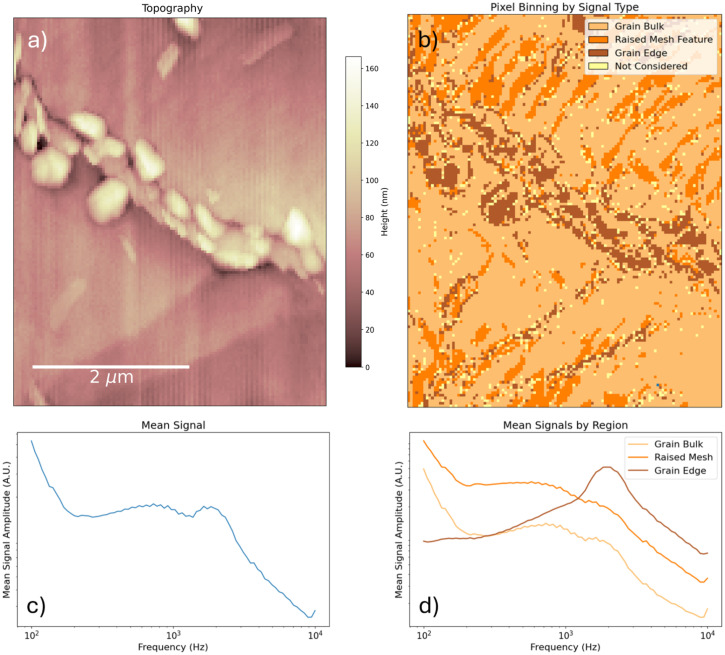
(a) Topography of a degraded triple-cation perovskite previously recrystallized under MA. Note faint meshlike texture atop the grain bulk as in Fig. 10 and a grain boundary populated with small raised granules. (b) Binning of measured spectra according to dominant NMF component. Note three kinds of signal, associated with the bulk grain, the raised mesh, and the grain boundary, respectively. (c) Mean spectrum over entire map. Note additional responses: a very low frequency component, a faint 150 Hz shoulder, a faint 200 Hz bump, and a possible 3 kHz shoulder. (d) Mean signal according to binning in (b).

Fig. 11 shows the topography of a scanned region, the spectrum binning by dominant NMF component, and the mean signal along with the mean by binning. In addition to the meshlike texture along the grain bulk, we note the apparent grain boundary is populated with many small, raised granulets, characteristic of advanced lead-iodide phase-segregation. The mean signal contains considerably more peaks than the pristine samples: there is a strong ultralow-frequency component, a small shoulder near 150 Hz, a faint peak at 200 Hz, and a possible shoulder at 3 kHz, in addition to the 1 kHz and 2 kHz peaks previously observed. NMF decomposition, binning by dominant NMF component, and averaging according to the binning, reveals three distinct signal types corresponding to (i) the raised meshlike texture atop the bulk grain, (ii) the bulk grain, and (iii) a grain boundary signal. The mesh and bulk signals do not vary much except in intensity – the mesh is about twice as bright as the grain bulk, as in the single-frequency scan. Meanwhile, the grain boundary presents a marked reduction in the 1 kHz signal (which persists only as a shoulder to the 2 kHz resonance), a strong enhancement of the 2 kHz resonance, and a lack of the secondary peaks previously mentioned. In particular, the ultralow-frequency response is absent in the grain boundary regions.

That the granules on the grain boundary are dominated by the 2 kHz resonance lends further credence to the notion that this is a signature of lead-iodide phase segregation. The appearance of additional peaks is puzzling – if anything, it suggests that the ionic landscape of a (triple-cation) lead-halide perovskite is finely dependent on its degradation-state or by the introduction of contaminants, and that perhaps additional mobile species are liberated over the course of film degradation or chemical treatment. If this is true, then special care must be taken in comparing the results of different studies – though the material may in principle match, the degradation-state may not.

## Conclusion

We have described a method to obtain information on the nanoscale distribution of ionic defect concentrations and mobilities across a lead-halide perovskite thin film. We observe a rich ionic landscape in triple-cation perovskites, including significant variation between grains and between grains and their boundaries. This variation has already been hinted at by macroscopic measurements of the perovskite dielectric response, which suggest a wide range of ionic mobilities across the sample,^56^ but here we observe it directly. This nanoscale heterogeneity along with its presumably fine dependence on film preparation, history, and environment, as well as the possible convolution of the nearby 2 kHz lead-iodide signature, may help explain the ongoing controversy regarding the activation energies of ionic defects in lead-halide perovskites. Of particular interest appears to be the sample's degradation-state, which may change not only over the course of but also due to measurement. While particularly pristine MAPI samples do not appear to locally phase-segregate into lead-iodide for at least a few weeks after fabrication, we have reason to suppose that lead-iodide plays a significant role in the ionic response of even a ‘fresh’ triple-cation sample – *i.e.* that there is no such thing as a ‘pristine’ triple-cation perovskite thin film.

The described method is quite general and lends itself to both (i) more detailed study of lead-halide perovskites, *i.e.* by varying temperature and bias voltage, by performing *in operando* measurements, or by monitoring the sample degradation over time, and (ii) the study of other materials heterogenous on the nanoscale and with significant ionic degrees of freedom, *e.g.* solid state electrolytes.

### Sample preparation

#### Triple-cation perovskite thin films

A 1.3 molar triple cation perovskite precursor was prepared by dissolving 73.4 mg PbBr_2_ and 507.1 mg PbI_2_ in 800 μL DMF and 200 μL DMSO (4 : 1 ratio). This mixture was heated to 100 °C for 30 min, then left to cool to room temperature. The cooled solution was then used to dissolve 22.4 mg MABr and 172 mg FAI. Finally, 53 μL of a 1.5-molar CsI solution in DMSO was added. This precursor was filtered in a 0.45 μm PTFE filter just before perovskite deposition.

ITO substrates (Lumtec, 15 Ω) were prepared with four subsequent 20 min ultrasonication steps in DI-water with detergent, pure DI water, acetone, and isopropanol respectively. Just before film deposition, a 20 min UV-ozone treatment was performed.

The triple cation perovskite films were spin-coated dynamically inside a nitrogen-filled glovebox. The spin-coating process comprised two steps: 10 s at 1000 rpm, then 50 s at 6000 rpm with an acceleration of 1000 rpm s^−1^. 20 s into the second step, 200 μL of CB were dropped as antisolvent. The samples were annealed at 120 °C for 10 min.

### MA-treatment

The pristine sample was put into a sealable treatment chamber and placed onto a hotplate. The sample temperature (73 °C, 82 °C, or 90 °C) was kept constant for the entire treatment. After 8 preparatory cleansing cycles, where the chamber was alternatingly flushed with nitrogen and then evacuated, the chamber was pumped one last time to 500 mbar. After these base conditions were established, 1.3 mmol methylamine gas, equivalent to a partial pressure of approximately 350 mbar, was let into the chamber. The perovskite film was exposed to this partial pressure for 25 s, leading to complete liquefaction of the film. Then, the total pressure was again reduced to 500 mbar, lowering the methylamine partial pressure to approximately 220 mbar and triggering recrystallization. Once the entire substrate was once again covered with the solid perovskite phase, the chamber could be flushed with nitrogen and the sample removed.

### MAPbI_3_ microcrystals

A Poly-[bis-(4-phenyl)-(2,4,6-trimethylphenyl)-amin] (PTAA) coating solution was made using 9 mg of PTAA and 6 mL of anhydrous toluene. 80 μL of coating solution was spin coated for 10 s at 1000 rpm and for 30 s at 5000 rpm on a 2.5 × 2.5 cm^2^ surface. Substrates were afterwards annealed for 10 minutes at 100 °C.

MAPbI_3_ single crystal thin films were fabricated by a confined inverse temperature method. Here, 10 μL of perovskite precursor solution consisting of 0.1 mmol methylammonium iodide (MAI) and 0.1 mmol PbI_2_ in 500 μL gamma-butyrolactone (GBL) was injected between an ITO-covered glass slide (bottom) and a PTAA-coated ITO-covered glass slide. In order to decrease the distance between the substrates and thus the thickness of crystals, two ferrite magnets (Y35 quality) were used to provide 1 MPa pressure between the slides. Then, the temperature was set to 90 °C and the setup remained untouched for 24 h. After that, the magnets were removed and the substrates were separated with a razor blade. The remaining solution was removed from the substrate by gently dabbing with a tissue, and the substrates were put onto the hot plate to be annealed at 90 °C for a few minutes. The crystals adhered to the PTAA-free ITO slide.

### Macroscopic MAPbI_3_ crystals

Free-standing MAPbI_3_ single crystals were also fabricated *via* inverse temperature crystallization method. 1 mmol MAI and 1 mmol PbI_2_ were dissolved in GBL at 60 °C. Then the solution was transferred into a Petri dish with a lid on top and the temperature was raised to 90 °C. Formed MAPbI_3_ single crystals were collected after 24 h.

## Other methods

### Single-frequency phase-modulated electric force microscopy

Instead of operating PM-DNS in a spectroscopic mode, it is possible to scan the surface (as in a conventional AFM measurement) with a fixed frequency of electrical stimulus. The resulting phase shift is subsequently demodulated over the course of the scan, yielding a map of the local capacitance gradient in addition to the topography. Similar electric force microscopy measurements can be performed in an amplitude-modulation mode,^37,38^ but not in the frequency-range accessible by phase-modulation as in PM-EFM.

### Piezoelectric force microscopy

Piezoelectric force microscopy (PFM) involves tracking the tip contact resonance across the surface of the sample.^58,69^ In practice, the sample piezoelectric response is stimulated by an electrical excitation at a fixed frequency, which is chosen to lie at or near the contact resonance for a set of twin domains. The amplitude and phase of the lateral oscillation of the cantilever induced by this stimulus will vary between sets of twins, resulting in the PFM contrast.

### Kelvin probe force microscopy

Kelvin Probe Force Microscopy (KPFM) is a method whereby the contact potential difference between tip and sample can be measured in feedback.^59^ There are many ways to perform KPFM measurements. In this study, we applied an electrical stimulus at a frequency equal to the difference of the cantilever's first two harmonics. This results in a mechanical oscillation of capacitive origin on the cantilever's second eigenmode, proportional to the capacitance gradient and most importantly the potential difference between the tip and sample. The inherent potential difference between tip and sample is the quantity of physical interest and gives us information regarding the local charge, work function contrast, and so on. By continually scanning the sample and using a feedback to sweep a bias that cancels the inherent contact potential difference, we are able to deduce the spatial variation in the CPD.

## Conflicts of interest

There are no conflicts to declare.

## Supplementary Material

NR-018-D5NR04126K-s001

## Data Availability

All experimental data supporting the findings in this study is available within the article. Additional raw data and analysis code can be provided by the corresponding author upon request. Supplementary information (SI) is available. Supplementary information (SI), including follow-up measurements and additional sample characterization, is available. See DOI: https://doi.org/10.1039/d5nr04126k.
