# Supplementary material for: Resolving ionic spectra of lead-halide perovskites to the nanometer
Source: Nanoscale. 2026 Mar 9;18(15):8263–77. doi: 10.1039/d5nr04126k (PMC12990059; doi:10.1039/d5nr04126k)
Supplement: NR-018-D5NR04126K-s001 [file NR-018-D5NR04126K-s001.pdf]

Supplementary Information (SI) for Nanoscale.
This journal is © The Royal Society of Chemistry 2026

Resolving Ionic Spectra of Lead-Halide Perovskites to the Nanometer
Supplementary Information

Lukas D. Ćavar,[†,‡] Emilia R. Schütz,[¶] Yenal Yalçinkaya,[¶,‡] Constantin Bach,[§] Carsten Deibel,[§] Lukas Schmidt-Mende,[¶] and Stefan A.L. Weber[*,†,‡,‖]

†*Department of Physics, Johannes Gutenberg University*

‡*Max Planck Institut für Polymerforschung*

¶*Department of Physics, Universität Konstanz*

§*Institut für Physik, Technische Universität Chemnitz*

‖*Institut für Photovoltaik, Universität Stuttgart*

E-mail: Stefan.Weber@ipv.uni-stuttgart.de

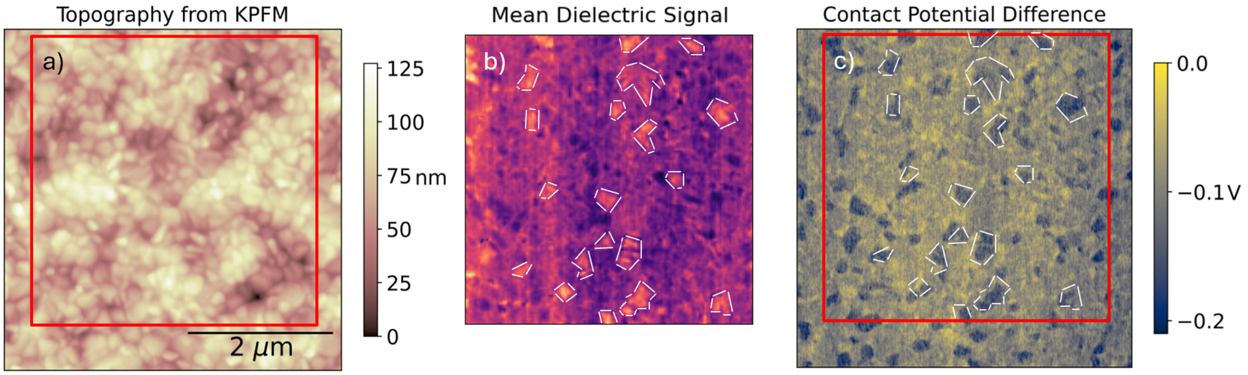

Figure S1: Comparison of PM-DNS and KPFM scans over the same region. a) Topography of scanned region obtained by KPFM, with red overlay showing the region of the PM-DNS scan. b) Mean dielectric signal obtained by PM-DNS, as in figure 1. Grains with enhanced signal intensity are outlined in white. c) Contact potential difference measured by KPFM. Hotspot grains from (b) are overlaid with minor adjustments to account for sample drift over the course of measurement. While not every CPD-enhanced grain presents an enhanced dielectric response, each bright dielectric grain is dark on the CPD.

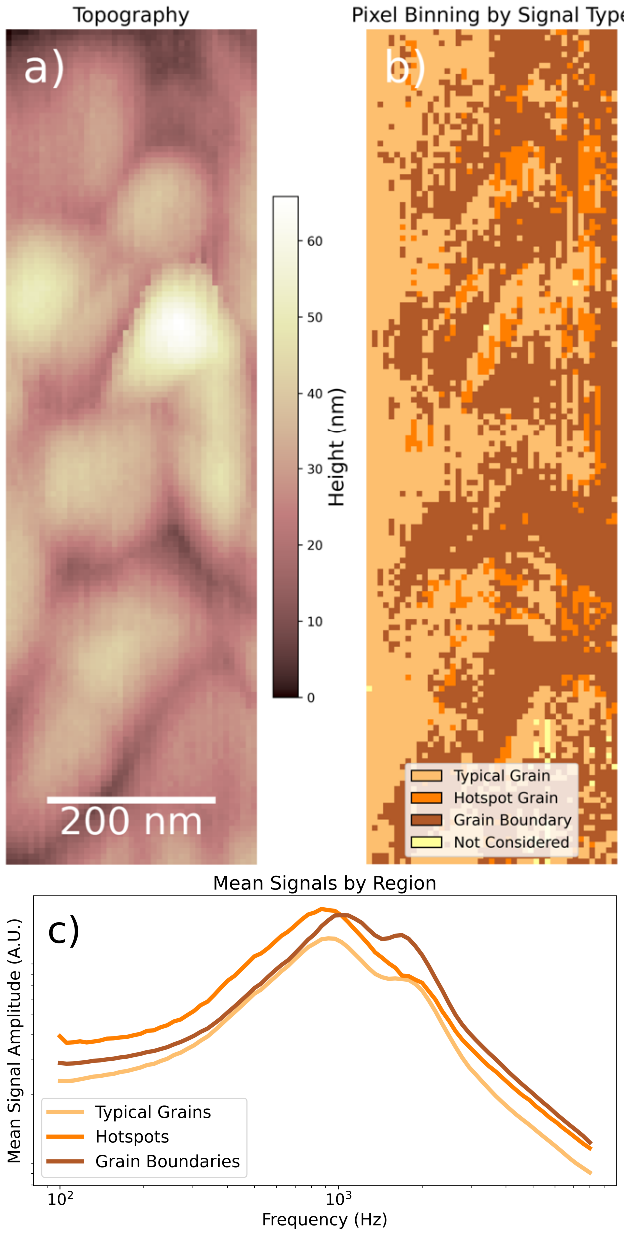

Figure S2: a) Granular topography of triple-cation perovskite thin film. b) Binning of pixels according to PM-DNS signal type– in particular, according to admixture of NMF components, as in figure 3. c) PM-DNS signal means by binning type.

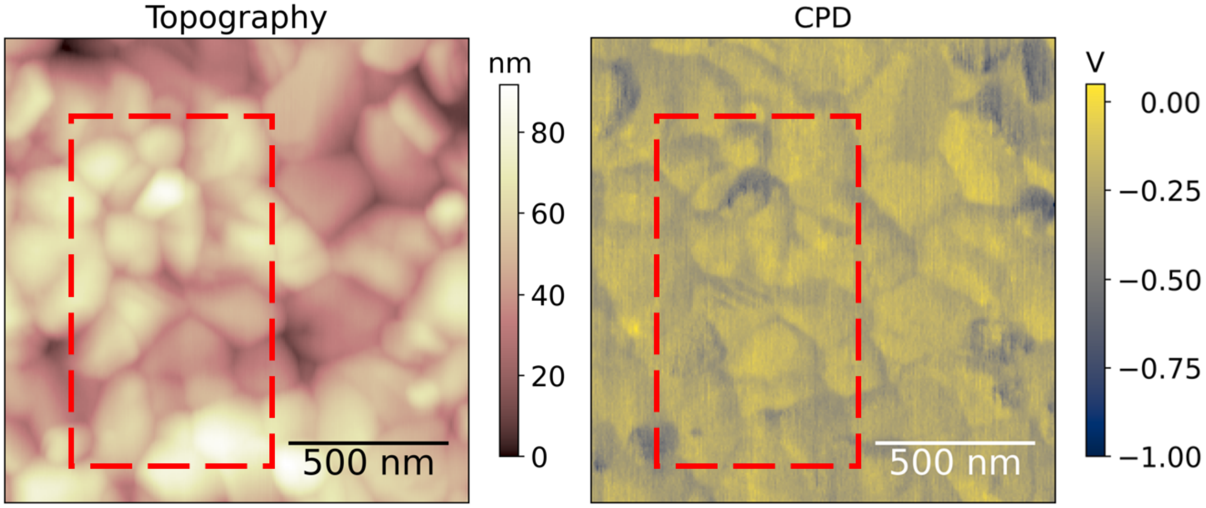

Figure S3: KPFM map containing same region scanned by PM-DNS in figure S2. a) Topography. b) Contact potential difference. Note CPD contrast appears somewhat different than in figure S1: the measurements are taken roughly a week apart, and we may attribute the difference in contrast to the onset of degradation or large-scale ionic redistribution over the course of the previous PM-DNS measurement. Note the large scale of CPD contrast here as compared to S1.

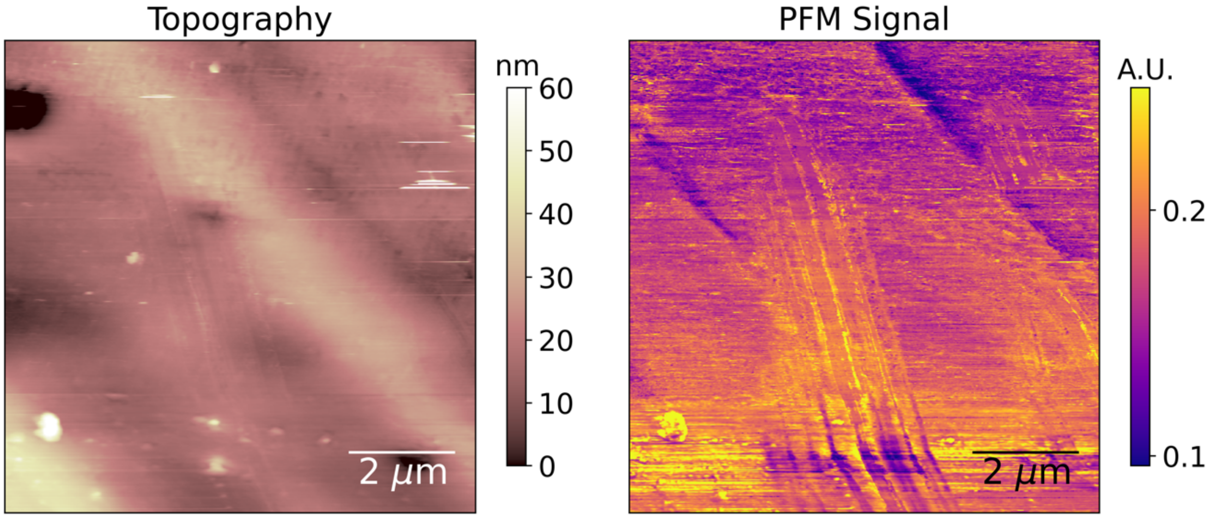

Figure S4: PFM of macroscopic MAPI pellet surface. a) Topography. b) PFM signal amplitude. Faint twinning signatures are visible in both measurements.

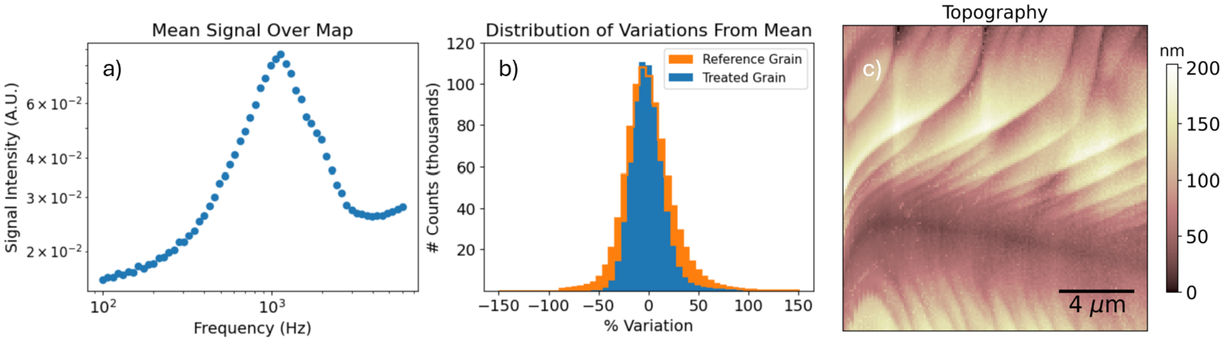

Figure S5: PM-DNS over a typical MA-treated triple-cation perovskite film treated at 90° C. a) Mean signal over entire map. b) Distribution of signal variations compared to reference grain depicted in figure 1. c) Topography. Note increased homogeneity of capacitive signal in the treated film, even in the presence of significant topographical contrast.

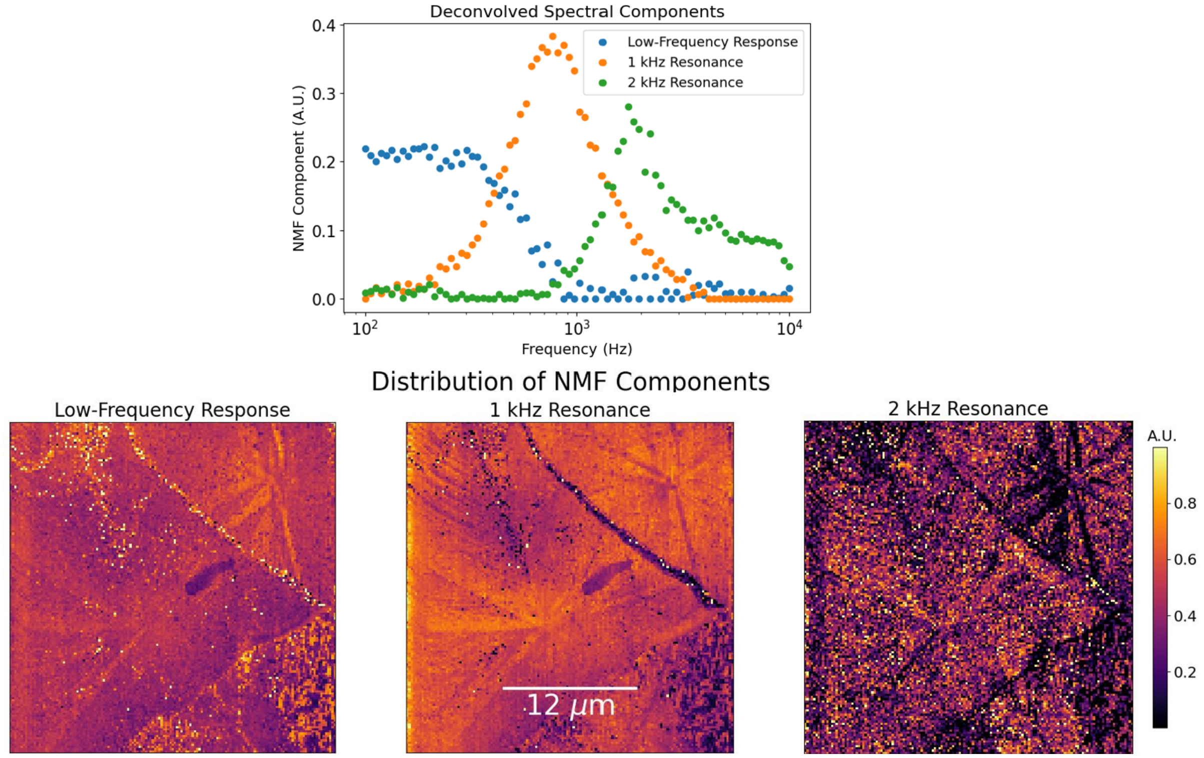

Figure S6: Principal components (top) and their distributions (bottom) extracted by NMF from the hyperspectra collected over the MA-treated grains depicted in figure 8. A contrast along the lines of the starlike projections is clearly visible.
